# Fitting and Testing Log-Linear Subpopulation Models with Known Support

**DOI:** 10.1007/s11336-023-09922-9

**Published:** 2023-06-14

**Authors:** David J. Hessen

**Affiliations:** Department of Methodology and Statistics, Utrecht University, Padualaan 14, PO Box 80.140, 3508 TC Utrecht, The Netherlands

**Keywords:** categorical variables, log-linear model, pseudo-likelihood, normalizing constant, Pearson chi-square test

## Abstract

In this paper, the support of the joint probability distribution of categorical variables in the total population is treated as unknown. From a general total population model with unknown support, a general subpopulation model with its support equal to the set of all observed score patterns is derived. In maximum likelihood estimation of the parameters of any such subpopulation model, the evaluation of the log-likelihood function only requires the summation over a number of terms equal to at most the sample size. It is made clear that the parameters of a hypothesized total population model are consistently and asymptotically efficiently estimated by the values that maximize the log-likelihood function of the corresponding subpopulation model. Next, new likelihood ratio goodness-of-fit tests are proposed as alternatives to the Pearson chi-square goodness-of-fit test and the likelihood ratio test against the saturated model. In a simulation study, the asymptotic bias and efficiency of maximum likelihood estimators and the asymptotic performance of the goodness-of-fit tests are investigated.

In practical applications of log-linear models (Agresti, 2013), the support of the joint probability distribution of the categorical variables in the population is usually assumed to be the set of all theoretically possible score patterns. Assuming the support to be equal to a proper subset of the set of all theoretically possible score patterns would result in a hybrid deterministic-probabilistic model. Assuming the support to be equal to the set of all theoretically possible score patterns makes the model completely probabilistic and is sensible in practice, where the true support is almost always unknown. However, when the number of categorical variables is large, the use of the all-inclusive support assumption has two well-known negative consequences.

The first negative consequence is that in maximum likelihood estimation of the model parameters the log-likelihood function cannot be evaluated efficiently because it requires the summation of a very large number of terms. To avoid this computational problem, maximum pseudo-likelihood methods have been proposed (Besag, 1975). Under regularity conditions, model parameters are consistently estimated using maximum pseudo-likelihood methods (Comets, 1992; Mase, 1995, 2000; Chatterjee, 2007; Mukherjee, 2016). A disadvantage of pseudo-likelihood methods, however, is that large sample sizes are needed to obtain stable parameter estimates (Geyer, 1991; Geyer and Thompson, 1992; Guyon and Künsch, 1992). Although regularization methods can be used in the case of a small sample size (Höfling and Tibshirani, 2009; Ravikumar et al., 2010), formal inference is not available in using pseudo-likelihood methods to estimate the parameters of a log-linear model. The pseudo-likelihood deviance of a log-linear model is not (asymptotically) chi-square distributed.

The second negative consequence is that Pearson’s asymptotic chi-square goodness-of-fit test and the usual likelihood ratio test of the assumed population model against the saturated model cannot be validly applied due to a too big difference in size between the assumed support and the set of all observed score patterns. If for many theoretically possible score patterns the sample frequency is zero, then the Pearson statistic and the likelihood ratio statistic are far from chi-square.

The all-inclusive support assumption, however, might have a third negative consequence. If the size of the unknown true support is much smaller than the size of the assumed all-inclusive support, then the model parameters might be estimated less accurately. Consider, for example, the situation of 20 categorical variables with each 3 categories, which is not uncommon in psychological testing, then the number of theoretically possible score patterns is $3^{20}=3,486,784,401$. It might be that many of these score patterns have zero probability. In that case, the model assigns positive probability to many score patterns that actually have zero probability. Such a misspecification of the support of the probability distribution of the categorical variables might have an adverse effect on the quality of the model parameter estimates.

In this paper, therefore, no assumption is made about the support of the joint probability distribution of the categorical variables. The position is adopted that the true support is unknown and not necessarily equal to the set of all theoretically possible score patterns. It is thought possible that some theoretically possible score patterns are not observable at all and are thus structurally missing. Although the exact support in the population is treated as unknown, it is obvious that the true support is a subset of the set of all theoretically possible score patterns and a superset of the set of all observed score patterns in the sample.

Fundamental to the present development is the observation that if observed score patterns are obtained by random sampling from a population, then they are also obtained by random sampling from any subpopulation defined by a superset of the set of all observed score patterns. The smallest such subpopulation is the subpopulation defined by the set of all observed score patterns. In this smallest subpopulation, the support of the joint probability distribution of the categorical variables is exactly equal to the set of all observed score patterns.

In this paper, therefore, from a general model for categorical variables in the total population with unknown support, a general model for the categorical variables in the subpopulation defined by the set of all observed score patterns is derived. Next, maximum likelihood estimation of the parameters of such subpopulation models is discussed. An advantage of maximum likelihood estimation of the parameters of any such subpopulation model is that the evaluation of the log-likelihood function only requires the summation of a number of terms equal to at most the sample size. In addition, it is made clear that the parameters of a hypothesized total population model are consistently and asymptotically efficiently estimated by the values that maximize the likelihood function of the corresponding subpopulation model.

Although Pearson’s chi-square test and the likelihood ratio test can now be used to test the goodness of fit of the subpopulation model (against a saturated subpopulation model), these tests are still not applicable if many of the observed score patterns have a low frequency. Therefore, for testing the goodness of fit of the subpopulation model, new likelihood ratio tests are proposed. One of these tests is a generalization of one of Andersen’s likelihood ratio tests for the Rasch model (Andersen, 1973). In addition to goodness-of-fit testing, some attention is paid to out of sample testing and cross-validation. To investigate the asymptotic bias and efficiency of maximum likelihood estimators of the parameters of the subpopulation model and the asymptotic performance of the goodness-of-fit tests, a simulation study is carried out.

## Models for Categorical Variables

### A General Model

Let ${\textbf{Y}}=(Y_{1},\ldots ,Y_{k})'$ be a random vector of *k* categorical variables and ${\textbf{y}}=(y_{1},\ldots ,y_{k})'$ be a realization, where $y_{i}\in \{0,1,\ldots ,m_{i}\}$, for $i\in \{1,2,\ldots ,k\}$. The set of all theoretically possible score patterns is the *k*-ary Cartesian product given by $A=\prod \nolimits _{i=1}^{k}\{0,1,\ldots ,m_{i}\}$. The number of elements of *A* is $\prod \nolimits _{i=1}^{k}(m_{i}+1)$ and exponentially increases with *k*.

The probability that ${\textbf{Y}}$ takes on the value ${\textbf{y}}$ for a randomly selected individual from the population is denoted by $P({\textbf{Y}}={\textbf{y}})$, for all ${\textbf{y}}\in A$, and equals the relative frequency of ${\textbf{y}}$ in the population. It is throughout assumed that the population and any subpopulation are infinite. In practice, it is usually assumed that $P({\textbf{Y}}={\textbf{y}})>0$, for all ${\textbf{y}}\in A$. It is, however, possible that $P({\textbf{Y}}={\textbf{y}})=0$, for some ${\textbf{y}}\in A$. Many probability models for categorical variables can now be generalized to the form given by

1 $$\begin{aligned} P({\textbf{Y}}={\textbf{y}})=\frac{{\textbf{1}}_{S}({\textbf{y}})exp \{f({\textbf{y}})\}}{\displaystyle \sum _{{\textbf{y}}\in S}exp \{f({\textbf{y}})\}},\ \ for all {\textbf{y}}\in A , \end{aligned}$$

where *S* is the unknown true support, that is, the smallest subset of *A* for which $\sum \nolimits _{{\textbf{y}}\in S}P({\textbf{Y}}={\textbf{y}})=1$, ${\textbf{1}}_{S}({\textbf{y}})$ is an indicator function, and $f({\textbf{y}})$ is a parametric function of ${\textbf{y}}$. Note that the support *S* is the subset of *A* that only contains all observable ${\textbf{y}}$, that is $S=\{{\textbf{y}}\in A\!\mid \!P({\textbf{Y}}={\textbf{y}})>0\}$.

The most general model of the form given by Eq. 1 is the saturated model. In the saturated model,

2 $$\begin{aligned} f({\textbf{y}})=\beta _{{\textbf{y}}},\ \ for all {\textbf{y}}\in S , \end{aligned}$$

where parameter $\beta _{{\textbf{y}}}$ is a real constant, for all ${\textbf{y}}\in S$. The arbitrary constraint $\sum \nolimits _{{\textbf{y}}\in S}\beta _{{\textbf{y}}}=0$ can be used for identification. The number of independent parameters of the saturated model equals the number of elements of *S* minus 1.

### Special Cases

Using dummy variables, the saturated model can be reparameterized in terms of all possible associations among the categorical variables $Y_{1},\ldots ,Y_{k}$. The reparameterized saturated model has many special cases. A whole family of hierarchical special cases is the set of models where associations of higher order than $r\in \{1,2,\ldots ,k-1\}$ are assumed to be absent. A well-known member of this family is the two-way association model. Let $x_{is}=1$ if $y_{i}=s$ and $x_{is}=0$ otherwise, for $s=1,\ldots ,m_{i}$, then the two-way association model in terms of dummy scores is given by

3 $$\begin{aligned} \begin{aligned} f({{\textbf {y}}})=\sum _{i}\varvec{\beta }_{i}'{{\textbf {x}}}_{i}+\sum _{i<j}{{\textbf {x}}}'_{i}{\varvec{\Sigma }}_{ij}{{\textbf {x}}}_{j}, \end{aligned} \end{aligned}$$

where $\varvec{\beta }_{i}=(\beta _{i1},\ldots ,\beta _{im_{i}})'$ is a vector of main effect parameters, ${\textbf{x}}_{i}=(x_{i1},\ldots ,x_{im_{i}})'$ is a vector of dummy scores, and $\varvec{\Sigma }_{ij}=[\,\sigma _{ijst}\,]$ is a $m_{i}\times m_{j}$ matrix of two-way association parameters, for all *i* and *j*. If $m_{i}=1$, for all *i*, then the two-way association model specializes to the Ising model (Ising, 1925). If $\varvec{\Sigma }_{ij}=\varvec{\Lambda }_{i}\varvec{\Psi }\varvec{\Lambda }'_{j}$, for all $i<j$, where $\varvec{\Lambda }_{i}$ is an $m_{i}\times q$ matrix, for all *i*, and $\varvec{\Psi }$ is a diagonal matrix of order $q\le k$, then the two-way association model specializes to the log-multiplicative association (LMA) model (Anderson and Yu, 2007; Anderson, 2013). If $\varvec{\Sigma }_{ij}={\textbf{s}}_{i}{\textbf{u}}_{i}'\varvec{\Psi }{\textbf{u}}_{j}{\textbf{s}}_{j}'$, for all $i<j$, where ${\textbf{s}}_{i}=(1,\ldots ,m_{i})'$ and ${\textbf{u}}_{i}=(u_{i1},\ldots ,u_{iq})'$ is a vector of fixed binary weights, for all *i*, and $\varvec{\Psi }$ is a $q\times q$ symmetric matrix, then the two-way association model specializes to the conditional multinormal partial credit model (Hessen, 2012). Another special case of the reparameterized saturated model, which is not a member of the discussed family of hierarchical models, is the extended partial credit model (Masters, 1982; Agresti, 1993). In the extended partial credit model, all associations of the same order are assumed to be equal so that,

4 $$\begin{aligned} f({\textbf{y}})=\displaystyle \sum \limits _{i}{\varvec{\beta }'}\!_{i}{\textbf{x}}_{i}+\displaystyle \sum \limits _{r=2}^{\varvec{k}}\sigma _{r}p_{r}({\textbf{1}}'{\textbf{y}}), \end{aligned}$$

where $\sigma _{r}$ is an *r*th-order association parameter, and $p_{r}({\textbf{1}}'{\textbf{y}})=(r!)^{-1}\prod \nolimits _{v=0}^{r-1}({\textbf{1}}'{\textbf{y}}-v)$ is an *r*th-order polynomial function of ${\textbf{1}}'{\textbf{y}}$. If $m_{i}=1$, for all *i*, then the extended partial credit model specializes to the extended Rasch model (Rasch, 1960, 1966; Tjur, 1982; Cressie and Holland, 1983; Follmann, 1988; Hessen, 2011).

Item response models in which the latent variables are assumed to follow a specific parametric distribution in the population of examinees are not special cases of the model in Eq. 1. These models do not require the summation of a very large number of terms and can be fitted to data using numerical integration techniques.

### A General Subpopulation Model

Any subset of the true support *S* defines a subpopulation of individuals. Let *B* be an arbitrary subset of *S*. The following theorem gives the general form of the probability that ${\textbf{Y}}={\textbf{y}}$, for all ${\textbf{y}}\in A$, for a randomly selected individual from the subpopulation defined by *B*.

#### Theorem 1

If the probability distribution of ${\textbf{Y}}$ in the total population is of the form given by Eq. 1, then the probability distribution of ${\textbf{Y}}$ in the subpopulation defined by *B* is equal to

5 $$\begin{aligned} P({\textbf{Y}}={\textbf{y}}\!\mid \!B)=\frac{{\textbf{1}}_{B}({\textbf{y}})exp \{f({\textbf{y}})\}}{\displaystyle \sum _{{\textbf{y}}\in B}exp \{f({\textbf{y}})\}},\ \ for all {\textbf{y}}\in A . \end{aligned}$$

#### Proof

If a score pattern is randomly sampled from the total population, then the probability of randomly sampling a score pattern from the set *B* is given by

6 $$\begin{aligned} P(B)=\sum _{{\textbf{y}}\in B}P({\textbf{Y}}={\textbf{y}}). \end{aligned}$$

Note that $P(B)=1$ if and only if $B=S$. Next, the joint probability distribution of ${\textbf{Y}}$ and *B* is given by

7 $$\begin{aligned} P({\textbf{Y}}={\textbf{y}},B)={\textbf{1}}_{B}({\textbf{y}})P({\textbf{Y}}={\textbf{y}}),\ \ \text {for all } {\textbf{y}}\in A, \end{aligned}$$

where ${\textbf{1}}_{B}({\textbf{y}})$ is an indicator function. Consequently, the conditional probability of ${\textbf{Y}}$ given *B* is given by

8 $$\begin{aligned} P({\textbf{Y}}={\textbf{y}}\!\mid \!B)=\frac{{\textbf{1}}_{B}({\textbf{y}})P({\textbf{Y}}={\textbf{y}})}{\displaystyle \sum _{{\textbf{y}}\in B}P({\textbf{Y}}={\textbf{y}})},\ \ for all {\textbf{y}}\in A . \end{aligned}$$

Substitution from Eq. 1 into Eq. 8 gives Eq. 5. This completes the proof.$\square$

Equation 5 is the general form of the conditional probability distribution of ${\textbf{Y}}$ given the subpopulation defined by *B*. It is important to see that this general form is implied by the total population model and that it contains as a special case the total population model because *S* is a subset of itself. This means that if the parametric function $f({\textbf{y}})$ holds true for the total population, then it also holds true for the subpopulation defined by *B*, with the same set of parameters. The general subpopulation model in Eq. 5, however, does not imply the total population model in Eq. 1. The general subpopulation model is more general than the total population model and only specializes to the total population model in Eq. 1 if also

9 $$\begin{aligned} P({\textbf{Y}}={\textbf{y}}\!\mid \!{\bar{B}})=\frac{{\textbf{1}}_{{\bar{B}}}({\textbf{y}})exp \{f({\textbf{y}})\}}{\displaystyle \sum _{{\textbf{y}}\in {\bar{B}}}exp \{f({\textbf{y}})\}},\ \ for all {\textbf{y}}\in A , \end{aligned}$$

where ${\bar{B}}=S\backslash B$ is the relative complement of *B* in *S*, and

10 $$\begin{aligned} P(B)=1-P({\bar{B}})=\frac{\sum \limits _{{\textbf{y}}\in B}exp \{f({\textbf{y}})\}}{\sum \limits _{{\textbf{y}}\in S}exp \{f({\textbf{y}})\}}, \end{aligned}$$

because then $P({\textbf{Y}}={\textbf{y}})=P({\textbf{Y}}={\textbf{y}}\!\mid \!B)P(B)+P({\textbf{Y}}={\textbf{y}}\!\mid \!{\bar{B}})P({\bar{B}})$ equals Eq. 1.

## Maximum Likelihood Estimation

To be able to practically apply a special case of the general subpopulation model in Eq. 5, the set *B* must be specified. In practice, it is usually assumed that $S=A$, so that *B* can be set equal to *A* or to a proper subset of *A*. Often, however, *S* is unknown and might be a proper subset of *A*. All that is known about *S* from the sample data is that it contains all observed score patterns. Let *O* be the set of all observed score patterns in the sample, that is, $O=\{{\textbf{y}}\in S\!\mid \!n_{{\textbf{y}}}>0\}$, where $n_{{\textbf{y}}}$ is the frequency of ${\textbf{y}}$ in the sample. Note that $O\subseteq S\subseteq A$ and that *O* defines the subpopulation of individuals with score patterns that have been observed in the sample. If $B=O$, then the subpopulation model in Eq. 5 has known support and no assumption is made about the support of the total population model in Eq. 1. So, in practice *B* can be set equal to *O* but if a practical situation gives rise to setting *B* equal to another proper subset of *A*, it stands to reason to choose *B* such that it at least includes *O*.

Since the parameters of the subpopulation model in Eq. 5 are exactly the same parameters as the parameters of the total population model in Eq. 1, the parameters of a hypothesized total population model can be estimated by fitting a subpopulation model defined by $B\supseteq O$. Let $n=\sum \nolimits _{{\textbf{y}}\in O}n_{{\textbf{y}}}$ be the size of the random sample from the total population and let $\varvec{\theta }$ be the vector of generic parameter values. Assuming independence of observations, the likelihood function under the model in Eq. 5 is given by

11 $$\begin{aligned} L_{B}=\kappa _{B}^{-n}exp \left\{ \sum _{{\textbf{y}}\in O}n_{{\textbf{y}}}f({\textbf{y}};\varvec{\theta })\right\} , \end{aligned}$$

where $\kappa _{B}=\sum \nolimits _{{\textbf{y}}\in B}exp \left\{ f({\textbf{y}};\varvec{\theta })\right\}$. The calculation of $\kappa _{B}$ requires the summation over as many terms as there are elements in *B*. If $B=A$, then $\kappa _{B}$ is the sum of $\prod \nolimits _{i=1}^{k}(m_{i}+1)$ terms and the computational efficiency rapidly decreases with *k* and $m_{1},\ldots ,m_{k}$. If $B=O$, then the calculation of $\kappa _{B}$ only requires summation over at most *n* terms.

Now, let $\varvec{\theta }_{0}$ be the vector of true parameter values and let $\hat{\varvec{\Theta }}_{B}$ be the vector of maximum likelihood estimators that maximizes $L_{B}$ with respect to $\varvec{\theta }$. It is well-known that under regularity conditions $\hat{\varvec{\Theta }}_{B}$ converges in probability to $\varvec{\theta }_{0}$, as $n\rightarrow \infty$, and $\sqrt{n}(\hat{\varvec{\Theta }}_{B}-\varvec{\theta }_{0})$ converges in distribution to the multivariate normal distribution with mean vector $\varvec{0}$ and covariance matrix ${\textbf{I}}^{-1}_{B}(\varvec{\theta }_{0})$, where

12 $$\begin{aligned} {\textbf{I}}_{B}(\varvec{\theta }_{0})=\left[ \sum _{{\textbf{y}}\in B}\frac{\partial P({\textbf{Y}}={\textbf{y}}\!\mid \!B)}{\partial \theta _{a}}\cdot \frac{\partial P({\textbf{Y}}={\textbf{y}}\!\mid \!B)}{\partial \theta _{b}}\cdot \frac{1}{P({\textbf{Y}}={\textbf{y}}\!\mid \!B)}\bigg |\varvec{\theta }_{0}\right] \end{aligned}$$

is the Fisher information matrix in the subpopulation defined by *B*, as $n\rightarrow \infty$. So, under regularity conditions, the asymptotic variance of the *a*th element of $\hat{\varvec{\Theta }}_{B}$ equals the Rao-Cramér lower bound $\frac{1}{n}\{{\textbf{I}}^{-1}_{B}(\varvec{\theta }_{0})\}_{a,a}$. Sufficient regularity conditions for the asymptotic properties of $\hat{\varvec{\Theta }}_{B}$ are: (1) any two different values of $\varvec{\theta }$ do not have the same $P({\textbf{Y}}={\textbf{y}}\!\mid \!B)$, for all ${\textbf{y}}\in B$ (theoretical identification), (2) *B* is independent of $\varvec{\theta }$, (3) $\varvec{\theta }_{0}$ is not on the boundary of the parameter space, (4) $P({\textbf{Y}}={\textbf{y}}\!\mid \!B)>0$ at $\varvec{\theta }_{0}$, for all ${\textbf{y}}\in B$, (5) $P({\textbf{Y}}={\textbf{y}}\!\mid \!B)$ has continuous first-order partial derivatives with respect to $\varvec{\theta }$ in a neighborhood of $\varvec{\theta }_{0}$, for all ${\textbf{y}}\in B$, and (6) the Jacobian matrix whose rows are given by $\partial P({\textbf{Y}}={\textbf{y}}\!\mid \!B)/\partial \varvec{\theta }$, for all ${\textbf{y}}\in B$, has full rank (equal to the length of $\varvec{\theta }$) at $\varvec{\theta }_{0}$. See also Agresti (2013, p. 592) and Lehmann (1999, pp. 499–501), for these regularity conditions, and Agresti (2013, ch. 16) and Lehmann and Casella (1998, ch. 6), for proofs. If sufficient regularity conditions are satisfied and $\hat{\varvec{\Theta }}_{B}$ is unique, then it is consistent, asymptotically normal, and asymptotically efficient given *B*. Note that if $B\supset S$ then regularity condition (4) is not satisfied. If, however, $B=O$, then regularity condition (4) is always satisfied.

If, under sufficient regularity conditions, *B* is a fixed subset of *S* and the sample size tends to infinity, then the elements of $\hat{\varvec{\Theta }}_{B}$ are in general consistent but only asymptotically efficient given *S* if $B=S$. The following theorem gives conditions under which the elements of $\hat{\varvec{\Theta }}_{B}$ are in general asymptotically efficient given *S*.

### Theorem 2

If *B* is a subset of *S* and includes the random set *O*, then, under sufficient regularity conditions, $\sqrt{n}(\hat{\varvec{\Theta }}_{B}-\varvec{\theta }_{0})$ converges in distribution to the multivariate normal distribution with mean vector $\varvec{0}$ and covariance matrix ${\textbf{I}}^{-1}_{S}(\varvec{\theta }_{0})$, as $n\rightarrow \infty$.

### Proof

If $n<|S|$, then *O* cannot be *S*, where |*S*| is the cardinality of *S*. On the other hand, if $n\ge |S|$, then *O* might be *S*. If $O=S$, then $B=S$ and $\hat{\varvec{\Theta }}_{B}=\hat{\varvec{\Theta }}_{S}$. If $\hat{\varvec{\Theta }}_{B}=\hat{\varvec{\Theta }}_{S}$, then $|\sqrt{n}(\hat{\varvec{\Theta }}_{B}-\varvec{\theta }_{0})-\sqrt{n}(\hat{\varvec{\Theta }}_{S}-\varvec{\theta }_{0})|=\sqrt{n}|\hat{\varvec{\Theta }}_{B}-\hat{\varvec{\Theta }}_{S}|<\varvec{\varepsilon }$, for all $\varvec{\varepsilon }>0$. As a consequence, $P(O=S)\le P(\hat{\varvec{\Theta }}_{B}=\hat{\varvec{\Theta }}_{S})\le P(\sqrt{n}|\hat{\varvec{\Theta }}_{B}-\hat{\varvec{\Theta }}_{S}|<\varvec{\varepsilon })$, where the elements of both $\hat{\varvec{\Theta }}_{B}$ and $\hat{\varvec{\Theta }}_{S}$ are discrete random variables. So, if $P(O=S)\rightarrow 1$, as $n\rightarrow \infty$, then the absolute difference between $\sqrt{n}(\hat{\varvec{\Theta }}_{B}-\varvec{\theta }_{0})$ and $\sqrt{n}(\hat{\varvec{\Theta }}_{S}-\varvec{\theta }_{0})$ converges in probability to zero. The set *O* equals *S* if and only if $n_{{\textbf{y}}}>0$, for all ${\textbf{y}}\in S$, where $n_{{\textbf{y}}}$ is a realization of random frequency $N_{{\textbf{y}}}$. If the joint probability that $N_{{\textbf{y}}}>0$, for all ${\textbf{y}}\in S$, tends to one, as $n\rightarrow \infty$, then $P(O=S)\rightarrow 1$. The joint probability that $N_{{\textbf{y}}}>0$, for all ${\textbf{y}}\in S$, is one minus the probability that $N_{{\textbf{y}}}=0$, for at least one ${\textbf{y}}\in S$, and therefore, this joint probability is greater than or equal to $1-\sum \nolimits _{{\textbf{y}}\in S}P(N_{{\textbf{y}}}=0)$. $N_{{\textbf{y}}}$ is a binomial random variable with binomial parameters *n* and $P({\textbf{Y}}={\textbf{y}})$, for all ${\textbf{y}}\in S$. It then follows that $P(O=S)\ge 1-\sum \nolimits _{{\textbf{y}}\in S}\{1-P({\textbf{Y}}={\textbf{y}})\}^{n}$, from which it can be seen that $P(O=S)\rightarrow 1$, as $n\rightarrow \infty$. So, the absolute difference between $\sqrt{n}(\hat{\varvec{\Theta }}_{B}-\varvec{\theta }_{0})$ and $\sqrt{n}(\hat{\varvec{\Theta }}_{S}-\varvec{\theta }_{0})$ converges in probability to zero, as $n\rightarrow \infty$. Consequently, $\sqrt{n}(\hat{\varvec{\Theta }}_{B}-\varvec{\theta }_{0})$ converges in distribution to the asymptotic distribution of $\sqrt{n}(\hat{\varvec{\Theta }}_{S}-\varvec{\theta }_{0})$, as $n\rightarrow \infty$, and this asymptotic distribution is multivariate normal with mean vector $\varvec{0}$ and covariance matrix ${\textbf{I}}^{-1}_{S}(\varvec{\theta }_{0})$. This completes the proof.$\square$

From the result in Theorem 2, it can now be concluded that under sufficient regularity conditions, the elements of $\hat{\varvec{\Theta }}_{B}$, where $O\subseteq B\subseteq S$, are in general asymptotically efficient as the sample size tends to infinity. From what follows, however, it is clear that the speed of convergence of the estimators in $\hat{\varvec{\Theta }}_{B}$ is lower than or equal to the speed of convergence of the estimators in $\hat{\varvec{\Theta }}_{S}$. First, note that it follows from Eq. 11 that

13 $$\begin{aligned} L_{B}=(\kappa _{B}/\kappa _{S})^{-n}L_{S}, \end{aligned}$$

where $\kappa _{B}/\kappa _{S}=P(B)$. It is obvious that if *P*(*B*) converges to 1, as $n\rightarrow \infty$, then $L_{B}$ converges to $L_{S}$, as $n\rightarrow \infty$. Now, since *P*(*B*) equals $\sum \nolimits _{{\textbf{y}}\in O}P({\textbf{Y}}={\textbf{y}})+\sum \nolimits _{{\textbf{y}}\in C}P({\textbf{Y}}={\textbf{y}})$, where *C* is the complement of *O* relative to *B*, *P*(*B*) converges to $\sum \nolimits _{{\textbf{y}}\in S}P({\textbf{Y}}={\textbf{y}})=1$, as $n\rightarrow \infty$, because $P(O=S)\rightarrow 1$, as $n\rightarrow \infty$. So, $L_{B}$ converges to $L_{S}$ and the values that maximize $L_{B}$ tend to the values that maximize $L_{S}$, as $n\rightarrow \infty$.

The general subpopulation model in Eq. 5 can be rewritten as the log-linear model

14 $$\begin{aligned} \ln {\mu _{{\textbf{y}}}}=\delta +f({\textbf{y}};\varvec{\theta }),\ \ for all {\textbf{y}}\in B, \end{aligned}$$

where $\mu _{{\textbf{y}}}=nP({\textbf{Y}}={\textbf{y}}\!\mid \!B)$ and $\delta =\ln (n/\kappa _{B})$. In the case of a standard log-linear subpopulation model, the maximum likelihood estimates of the parameters of the model can be obtained using iterative weighted least squares (Charnes et al., 1976). The Broyden–Fletcher–Goldfarb–Shanno (BFGS) algorithm (Fletcher, 1987), on the other hand, can be used to obtain the maximum likelihood estimates of the parameters of both standard and non-standard log-linear subpopulation models.

## Goodness-of-Fit Tests

### Pearson’s Chi-Square and Likelihood Ratio

The observed frequencies of score patterns in *B* are realizations of random frequencies that can be assumed to jointly follow a conditional multinomial distribution given *B* with parameters *n* and $P({\textbf{Y}}={\textbf{y}}\!\mid \!B)$, for all ${\textbf{y}}\in B$. Therefore, to assess the goodness of fit of the log-linear model of interest in the subpopulation defined by fixed set *B*, where $O\subseteq B\subseteq S$, the Pearson chi-squared test might be appropriate. The observed value of Pearson’s statistic for testing the model in Eq. 5 is given by

15 $$\begin{aligned} \sum _{{\textbf{y}}\in B}\frac{(n_{{\textbf{y}}}-n{\hat{\pi }}_{{\textbf{y}}|B})^{2}}{n{\hat{\pi }}_{{\textbf{y}}|B}}, \end{aligned}$$

where ${\hat{\pi }}_{{\textbf{y}}|B}$, for all ${\textbf{y}}\in B$, is the estimate of the conditional probability in Eq. 5, under a special case. Under any identified model in the subpopulation defined by the fixed set *B*, the Pearson statistic is asymptotically chi-square distributed with the degrees of freedom equal to $|B|-1-e$, where *e* is the number of free parameters in the hypothesized subpopulation model.

In the case of fixed set *B*, the asymptotic sampling distribution of the Pearson statistic in Eq. 15 can be conceived of as follows. Imagine that infinitely many random samples of size *n* have been drawn from the total population. The set *B* can be selected in advance or a superset of the set of all observed score patterns in one of the random samples is taken as *B*. Observed score patterns that are not in the selected fixed set *B* are removed from all random samples. New score patterns can be randomly sampled from the total population, and if these new randomly sampled score patterns belong to *B*, they can be added to the random samples until all samples are of size *n*. Subsequently, the same hypothesized special case of the general subpopulation model in Eq. 5 is fitted to all these random samples of size *n*. Next, the observed value of the Pearson statistic in Eq. 15 is calculated for all these random samples. The chi-square distribution with its degrees of freedom equal to $|B|-1-e$ is now the asymptotic sampling distribution to which the frequency distribution of the observed sample values of the Pearson statistic approaches, as *n* tends to infinity.

An alternative goodness-of-fit test is the likelihood ratio test of the subpopulation model against the saturated subpopulation model. The likelihood function for the saturated subpopulation model is given by

16 $$\begin{aligned} L^{*}_{B}=\prod _{{\textbf{y}}\in B}\pi _{{\textbf{y}}|B}^{n_{{\textbf{y}}}}, \end{aligned}$$

where $\pi _{{\textbf{y}}|B}={\textbf{1}}_{B}({\textbf{y}})exp (\beta _{{\textbf{y}}})/\sum _{{\textbf{y}}\in B}exp (\beta _{{\textbf{y}}})$, for all ${\textbf{y}}\in A$. The maximum likelihood estimate of $\pi _{{\textbf{y}}|B}$ is $n_{{\textbf{y}}}/n$, for all ${\textbf{y}}\in B$. So, the maximum of the log-likelihood function under the saturated subpopulation model is given by

17 $$\begin{aligned} {\hat{l}}^{*}_{B}=\sum _{{\textbf{y}}\in B}n_{{\textbf{y}}}\ln {n_{{\textbf{y}}}}-n\ln {n}. \end{aligned}$$

The observed value of the likelihood ratio statistic is then given by

18 $$\begin{aligned} 2\!\left( {\hat{l}}^{*}_{B}-\ln {{\hat{L}}_{B}}\right) \!, \end{aligned}$$

where ${\hat{L}}_{B}$ is the maximum of the likelihood function under the particular subpopulation model fitted to the data. Under any identified model for the subpopulation defined by the fixed set *B*, the likelihood ratio statistic in Eq. 18 has the same asymptotic sampling distribution as the Pearson chi-square statistic in Eq. 15.

In the case of random set *B*, we have the following result for the Pearson and likelihood ratio statistics. Since $P(O=S)\rightarrow 1$, as $n\rightarrow \infty$, also $P(B=S)\rightarrow 1$, as $n\rightarrow \infty$, and each of the two statistics given *B* converges in probability to the corresponding statistic given *S*. As a consequence, the distribution of each statistic given *B* converges to the distribution of the corresponding statistic given *S*. Since the limiting distribution of each statistic given *S* is a chi-square distribution with $|S|-1-e$ degrees of freedom, both statistics given *B* converge in distribution to the chi-square distribution with $|S|-1-e$ degrees of freedom.

### New Likelihood Ratio Tests

If many observed score pattern frequencies are low, then the Pearson statistic and the likelihood ratio statistic are known to be far from chi-squared and thus not applicable. For such a practical situation, the likelihood ratio tests that are presented in this section might be more useful.

To be able to test a special case of the general subpopulation model in Eq. 5 using a likelihood ratio test, a more general model is needed. In what follows, two generalizations of the subpopulation model in Eq. 5 are presented and it is shown how they can be used in a likelihood ratio test of a particular subpopulation model. The two generalizations are obtained by imposing restrictions on a reformulation of the general form of the likelihood function $L_{B}$. The general form of $L_{B}$ is given by

19 $$\begin{aligned} L_{B}=\prod _{{\textbf{y}}\in O}\{P({\textbf{Y}}={\textbf{y}}\!\mid \!B)\}^{n_{{\textbf{y}}}}=\frac{\prod \limits _{{\textbf{y}}\in O}\{P({\textbf{Y}}={\textbf{y}})\}^{n_{{\textbf{y}}}}}{\displaystyle \left\{ \sum _{{\textbf{y}}\in B}P({\textbf{Y}}={\textbf{y}})\right\} ^{\!n}}. \end{aligned}$$

Now, in the following theorem, a reformulation of the general form of $L_{B}$ is given in terms of the elements of a partition of the fixed set *B*.

#### Theorem 3

If $B_{1},\ldots ,B_{g}$ are the elements of a partition of *B*, then the general likelihood function in Eq. 19 equals

20 $$\begin{aligned} L_{B}=\prod _{p=1}^{g}\prod _{{\textbf{y}}\in O_{p}}\{P({\textbf{Y}}={\textbf{y}}\!\mid \!B_{p})P(B_{p}\!\mid \!B)\}^{n_{{\textbf{y}}}}, \end{aligned}$$

where $O_{1},\ldots ,O_{g}$ are the elements of a partition of *O* such that $O_{p}\subseteq B_{p}$, for all *p*, and

21 $$\begin{aligned} P({\textbf{Y}}={\textbf{y}}\!\mid \!B_{p})=\frac{P({\textbf{Y}}={\textbf{y}},B_{p})}{P(B_{p})}=\frac{{\textbf{1}}_{B_{p}}({\textbf{y}})P({\textbf{Y}}={\textbf{y}})}{\sum \limits _{{\textbf{y}}\in B_{p}}P({\textbf{Y}}={\textbf{y}})},\ \ for all {\textbf{y}}\in A, \end{aligned}$$

is the conditional probability distribution of ${\textbf{Y}}$ given the subpopulation defined by $B_{p}$, for all *p*, and

22 $$\begin{aligned} P(B_{p}\!\mid \!B)=\frac{\sum _{{\textbf{y}}\in B_{p}}P({\textbf{Y}}={\textbf{y}})}{\sum _{{\textbf{y}}\in B}P({\textbf{Y}}={\textbf{y}})},\ \ for all { p}. \end{aligned}$$

#### Proof

Substitution from $\prod \nolimits _{{\textbf{y}}\in O}\{P({\textbf{Y}}={\textbf{y}})\}^{n_{{\textbf{y}}}}=\prod \nolimits _{p=1}^{g}\prod \nolimits _{{\textbf{y}}\in O_{p}}\{{\textbf{1}}_{B_{p}}({\textbf{y}})P({\textbf{Y}}={\textbf{y}})\}^{n_{{\textbf{y}}}}$ and $n=\sum \nolimits _{p=1}^{g}\sum \nolimits _{{\textbf{y}}\in O_{p}}n_{{\textbf{y}}}$ into the right-hand side of Eq. 19 yields

23 $$\begin{aligned} L_{B}=\prod \limits _{p=1}^{g}\prod \limits _{{\textbf{y}}\in O_{p}}\left\{ \frac{{\textbf{1}}_{B_{p}}({\textbf{y}})P({\textbf{Y}}={\textbf{y}})}{\sum _{{\textbf{y}}\in B}P({\textbf{Y}}={\textbf{y}})}\right\} ^{n_{{\textbf{y}}}}. \end{aligned}$$

Next, multiplying both the numerator and the denominator in the right-hand side of Eq. 23 by $\sum \nolimits _{{\textbf{y}}\in B_{p}}P({\textbf{Y}}={\textbf{y}})$ gives Eq. 20. This completes the proof.$\square$

The elements of the partition can be chosen in many different ways. One simple way is to randomly assign score patterns in *B* to $B_{1},\ldots ,B_{g}$. Alternatively, the elements of the partition can be selected on the basis of the values of a particular function of ${\textbf{y}}$. For instance, one general choice of the *p*th element is $B_{p}=\{{\textbf{y}}\in B\!\mid \!{\textbf{z}}={\textbf{c}}_{p}\}$, for $p=1,\ldots ,g$, where the elements of the vector ${\textbf{z}}$ are the elements of a nonempty subset of $\{y_{1},y_{2},\ldots ,y_{k}\}$ and ${\textbf{c}}_{1},{\textbf{c}}_{2},\ldots ,{\textbf{c}}_{g}$ are the distinct possible values of ${\textbf{z}}$. Example choices of ${\textbf{z}}$ are $y_{2}$ and $(y_{1},y_{3})'$. A second general choice of the *p*th element is $B_{p}=\{{\textbf{y}}\in B\!\mid \!{\textbf{w}}'{\textbf{y}}=t_{p}\}$, for $p=1,\ldots ,g$, where each element of the vector ${\textbf{w}}$ is an element of $\{0,1\}$ and $t_{1},t_{2},\ldots ,t_{g}$ are the distinct possible values of ${\textbf{w}}'{\textbf{y}}$. Example choices of ${\textbf{w}}$ are $(1,1,0,\ldots ,0)'$, in which case ${\textbf{w}}'{\textbf{y}}=y_{1}+y_{2}$, and the vector of ones, in which case ${\textbf{w}}'{\textbf{y}}=\sum _{i}y_{i}$. A third general choice of the *p*th element is $B_{p}=\{{\textbf{y}}\in B\!\mid \!d_{p-1}\le {\textbf{w}}'{\textbf{y}}<d_{p}\}$, for $p=1,\ldots ,g$, where $d_{0},d_{1},\ldots ,d_{g}$ are percentiles of ${\textbf{w}}'{\textbf{y}}$ in *B*.

A first generalization of the general subpopulation model in Eq. 5 is now given by the model in which it is assumed that

24 $$\begin{aligned} P({\textbf{Y}}={\textbf{y}}\!\mid \!B_{p})=\frac{{\textbf{1}}_{B_{p}}({\textbf{y}})exp \{f({\textbf{y}};\varvec{\theta }_{p})\}}{\sum \limits _{{\textbf{y}}\in B_{p}}\!\text {exp}\{f({\textbf{y}};\varvec{\theta }_{p})\}},\ \ \text {for all }{\textbf{y}}\in A \text { and } p, \end{aligned}$$

where $P(B_{p}\!\mid \!B)$ is unrestricted and treated as a parameter, for all *p*. This generalization specializes to the subpopulation model in Eq. 5, if $\varvec{\theta \!}_{p}=\varvec{\theta }$ and

25 $$\begin{aligned} P(B_{p}\!\mid \!B)=\frac{\sum _{{\textbf{y}}\in B_{p}}exp \{f({\textbf{y}};\varvec{\theta })\}}{\sum _{{\textbf{y}}\in B}exp \{f({\textbf{y}};\varvec{\theta })\}},\ \ for all { p} . \end{aligned}$$

Under this generalization, the likelihood function in Eq. 20 equals

26 $$\begin{aligned} L_{B}=\prod _{p=1}^{g}L_{p}\cdot \left\{ P(B_{p}\!\mid \!B)\right\} ^{n_{p}}, \end{aligned}$$

where $$n_{p}=\sum _{{\textbf{y}}\in O_{p}}n_{{\textbf{y}}}$$ and

27 $$\begin{aligned} L_{p}=\prod \limits _{{\textbf{y}}\in O_{p}}\!\left\{ P({\textbf{Y}}={\textbf{y}}\!\mid \!B_{p})\right\} ^{n_{{\textbf{y}}}}=exp \left\{ \sum \limits _{{\textbf{y}}\in O_{p}}n_{{\textbf{y}}}f({\textbf{y}};\varvec{\theta }_{p})\right\} \!/\!\left[ \sum _{{\textbf{y}}\in B_{p}}exp \{f({\textbf{y}};\varvec{\theta }_{p})\}\right] ^{\!n_{p}} \end{aligned}$$

is the likelihood function for the subpopulation defined by $B_{p}$, for all *p*. The values that maximize $L_{p}$, for all *p*, can be found by fitting the log-linear model

28 $$\begin{aligned} \ln {\mu _{{\textbf{y}}}}=\delta _{p}+f({\textbf{y}};\varvec{\theta }_{p}),\ \ for all {\textbf{y}}\in B and { p}, \end{aligned}$$

where $\mu _{{\textbf{y}}}=n_{p}P({\textbf{Y}}={\textbf{y}}\!\mid \!B_{p})$ and $\delta _{p}=\ln \!\left[ n_{p}/\sum _{{\textbf{y}}\in B_{p}}exp \{f({\textbf{y}};\varvec{\theta }_{\!p})\}\right]$. Note that the likelihood function in Eq. 26 under the first generalization is a product of the likelihood functions $L_{1},\ldots ,L_{g}$ and the likelihood function $\prod _{p=1}^{g}\left\{ P(B_{p}\!\mid \!B)\right\} ^{n_{p}}$. Since none of these likelihood functions depends on the parameters of the other likelihood functions, the likelihood function in Eq. 26 under the first generalization can be maximized by maximizing each of these likelihood functions separately. This independence of likelihood functions can easily be seen by taking the first derivative of the logarithm of the right-hand side of Eq. 26 with respect to either $\varvec{\theta }_{p}$ or the vector $\varvec{\pi }_{B}=\{P(B_{1}\!\mid \!B),\ldots ,P(B_{g}\!\mid \!B)\}'$. The resulting gradient is independent of all other parameter vectors. The conditional probabilities $P(B_{1}\!\mid \!B),\ldots ,P(B_{g}\!\mid \!B)$ are recognized as multinomial probabilities and then it follows from a well known result for multinomial probabilities that the maximum likelihood estimate of $P(B_{p}\!\mid \!B)$ equals $n_{p}/n$, for all *p*.

Let $\hat{\varvec{\Theta }}_{p}$ be the vector of maximum likelihood estimators that maximizes $L_{p}$ with respect to $\varvec{\theta }$, for all *p*, and let the vector ${\textbf{N}}/n=(N_{1}/n,\ldots ,N_{g}/n)'$ be the vector of maximum likelihood estimators of the elements of $\varvec{\pi }_{B}$. Under regularity conditions, the elements of $\hat{\varvec{\Theta }}_{p}$, for all *p*, and ${\textbf{N}}/n$ are consistent estimators, $\sqrt{n_{p}}(\hat{\varvec{\Theta }}_{p}-\varvec{\theta }_{0})$ is asymptotically multivariate normal with mean vector ${\textbf{0}}$ and covariance matrix ${\textbf{I}}^{-1}_{p}(\varvec{\theta }_{0})$ (the inverse of the Fisher information matrix in the subpopulation defined by $B_{p}$), for all *p*, and $\sqrt{n}({\textbf{N}}/n-\varvec{\pi }_{B})$ is asymptotically multivariate normal with mean vector ${\textbf{0}}$ and covariance matrix $\varvec{\Delta }_{B}=diag (\varvec{\pi }_{B})-\varvec{\pi }_{B}\varvec{\pi }'_{B}$. Since the vectors $\hat{\varvec{\Theta }}_{1},\ldots ,\hat{\varvec{\Theta }}_{g}$ and ${\textbf{N}}/n$ are mutually independent, their joint distribution is asymptotically multivariate normal with mean vector ${\textbf{0}}$ and a block diagonal covariance matrix, where the main-diagonal blocks are ${\textbf{I}}^{-1}_{1}(\varvec{\theta }_{0}),\ldots ,{\textbf{I}}^{-1}_{g}(\varvec{\theta }_{0})$, and $\varvec{\Delta }_{B}$. According to a fundamental result by Wilks (1938), it then follows that under a hypothesized regular special case of the subpopulation model in Eq. 5, the value given by

29 $$\begin{aligned} -2\ln {\left\{ \displaystyle \frac{{\hat{L}}_{B}}{\prod \limits _{p=1}^{g}{\hat{L}}_{p}\cdot \!\left( \frac{n_{p}}{n}\right) ^{n_{p}}}\right\} }, \end{aligned}$$

where ${\hat{L}}_{B}$ is the maximum of $L_{B}$ in Eq. 11 and ${\hat{L}}_{p}$ is the maximum of $L_{p}$ in Eq. 27, for all *p*, is the observed sample value of a random variable having an asymptotic chi-square distribution with $(g-1)(e+1)$ degrees of freedom. Note that the denominator in Formula 29 is the maximum of the likelihood function in Eq. 26 under the first generalization of the hypothesized subpopulation model, and the numerator is the maximum of this same likelihood function if the parameter space is restricted by the hypothesized subpopulation model.

A second generalization of the general subpopulation model in Eq. 5 is the special case of the first generalization for which $\varvec{\theta }_{p}=\varvec{\theta }$, for all *p*, so that

30 $$\begin{aligned} P({\textbf{Y}}={\textbf{y}}\!\mid \!B_{p})=\frac{{\textbf{1}}_{B_{p}}({\textbf{y}})exp \{f({\textbf{y}};\varvec{\theta })\}}{\sum \limits _{{\textbf{y}}\in B_{p}}\!exp \{f({\textbf{y}};\varvec{\theta })\}},\ \ for all {\textbf{y}}\in A and { p}, \end{aligned}$$

and $P(B_{p}\!\mid \!B)$ is again unrestricted, for all *p*. This generalization specializes to the general subpopulation model in Eq. 5, if Eq. 25 is satisfied. Under this generalization, the likelihood function in Eq. 20 is given by

31 $$\begin{aligned} L_{B}=L_{C}\prod _{p=1}^{g}\!\left\{ P(B_{p}\!\mid \!B)\right\} ^{n_{p}}, \end{aligned}$$

where

32 $$\begin{aligned} L_{C}=\prod \limits _{p=1}^{g}L_{p}=exp \left\{ \sum \limits _{{\textbf{y}}\in B}n_{{\textbf{y}}}f({\textbf{y}};\varvec{\theta })\right\} \!\prod \limits _{p=1}^{g}\!\left[ \sum _{{\textbf{y}}\in B_{p}}exp \{f({\textbf{y}};\varvec{\theta })\}\right] ^{\!-n_{p}} \end{aligned}$$

is a total likelihood function for the subpopulations defined by $B_{1},\ldots ,B_{g}$. The values that maximize $L_{C}$ can be found by fitting the log-linear model

33 $$\begin{aligned} \ln {\mu _{{\textbf{y}}}}=\delta _{p}+f({\textbf{y}};\varvec{\theta }),\ \ for all {\textbf{y}}\in B and { p}, \end{aligned}$$

where $$\mu _{{\textbf{y}}}=n_{p}P({\textbf{Y}}={\textbf{y}}\!\mid \!B_{p})$$ and $$\delta _{p}=\ln \!\left[ n_{p}/\sum _{{\textbf{y}}\in B_{p}}exp \{f({\textbf{y}};\varvec{\theta })\}\right] $$.

Let $\hat{\varvec{\Theta }}_{C}$ be the vector of maximum likelihood estimators that maximizes $L_{C}$ with respect to $\varvec{\theta }$. Under regularity conditions, the elements of $\hat{\varvec{\Theta }}_{C}$ are consistent estimators and $\sqrt{n}(\hat{\varvec{\Theta }}_{C}-\varvec{\theta }_{0})$ is asymptotically multivariate normal with mean vector ${\textbf{0}}$ and covariance matrix ${\textbf{I}}^{-1}_{C}(\varvec{\theta }_{0})$, where

$$\begin{aligned}{\textbf{I}}_{C}(\varvec{\theta }_{0})=-E\!\left[ \frac{\partial ^{2}\ln L_{C}}{\partial \varvec{\theta }^{2}}\bigg |\varvec{\theta }_{0}\right] .\end{aligned}$$

Since the vectors $\hat{\varvec{\Theta }}_{C}$ and ${\textbf{N}}/n$ are mutually independent, their joint distribution is asymptotically multivariate normal with mean vector ${\textbf{0}}$ and a block diagonal covariance matrix, where the main-diagonal blocks are ${\textbf{I}}^{-1}_{C}(\varvec{\theta }_{0})$ and $\varvec{\Delta }_{B}$. According to the fundamental result by Wilks (1938), it now follows that under a hypothesized regular special case of the general subpopulation model in Eq. 5, the value given by

34 $$\begin{aligned} -2\ln {\left\{ \frac{{\hat{L}}_{B}}{{\hat{L}}_{C}\prod \limits _{p=1}^{g}\!\left( \frac{n_{p}}{n}\right) ^{n_{p}}}\right\} }, \end{aligned}$$

where ${\hat{L}}_{B}$ is again the maximum of $L_{B}$ in Eq. 11 and ${\hat{L}}_{C}$ is the maximum of $L_{C}$ in Eqs. 31 and 32, is the observed sample value of a random variable having an asymptotic chi-square distribution with $g-1$ degrees of freedom. Note that the denominator in Formula 34 is the maximum of the likelihood function in Eq. 31 under the second generalization of the hypothesized model, and the numerator is the maximum of this same likelihood function if the parameter space is restricted by the hypothesized subpopulation model.

Instead of a hypothesized special case of the general subpopulation model in Eq. 5, its second generalization in Eq. 30 can be tested against its first generalization in Eq. 24 using a likelihood ratio test. According to the fundamental result by Wilks (1938), it follows that under the second generalization of the hypothesized regular special case, the value given by

35 $$\begin{aligned} -2\ln {\!\left( \frac{{\hat{L}}_{C}}{\prod \limits _{p=1}^{g}{\hat{L}}_{p}}\right) } \end{aligned}$$

is the observed sample value of a random variable having an asymptotic chi-square distribution with $(g-1)e$ degrees of freedom. Note that the likelihood ratio in Formula 35 is obtained by dividing the maximum of the likelihood function in Eq. 31 by the maximum of the likelihood in Eq. 26. The denominator in Formula 35 is the maximum of the likelihood function in Eq. 26 under the first generalization of the hypothesized subpopulation model, and the numerator is the maximum of this same likelihood function if the parameter space is restricted by the second generalization of the hypothesized subpopulation model. If $B=A$ and $m_{i}=1$, for all *i*, $f({\textbf{y}})$ is given by Eq. 4, and $B_{p}=\{{\textbf{y}}\in A\!\mid \!{\textbf{1}}'{\textbf{y}}=p\}$, for $p=1,\ldots ,k-1$, then the likelihood ratio test for which the value of the observed sample statistic is given by Formula 35 coincides with one of Andersen’s likelihood ratio tests for the Rasch model (Andersen, 1973; Rasch, 1960, 1966).

In the case of random *B*, the three likelihood ratio statistics with the observed sample values in Formulas 29, 34, and 35 have the same limiting chi-square distributions as in the case of fixed *B* because the degrees of freedom of these limiting chi-square distributions are independent of |*B*| and only depend on *g* and *e*.

### Out-of-Sample Testing and Cross-Validation

Let *O* be the set of observed score patterns in a training sample of size *n* and let $O_{0}$ be the set of observed score patterns in a validation sample of size $n_{0}$. The training sample is assumed to be randomly sampled from the subpopulation defined by $B\supseteq O$ and the validation sample is assumed to be randomly sampled from the subpopulation defined by $B_{0}\supseteq O_{0}$. Let $n_{{\textbf{y}}}$ be the observed frequency of score pattern ${\textbf{y}}$ in the training sample, and let $n_{{\textbf{y}}0}$ be the observed frequency of ${\textbf{y}}$ in the validation sample. Let $\hat{\varvec{\theta }}$ be the value of $\varvec{\theta }$ that maximizes the likelihood function $L_{B}$ in the training sample, then the predicted frequency of score pattern ${\textbf{y}}$ in the validation sample, is given by

36 $$\begin{aligned} {\hat{n}}_{{\textbf{y}}}=n_{0}\frac{exp \{f({\textbf{y}};\hat{\varvec{\theta }})\}}{\displaystyle \sum _{{\textbf{y}}\in B_{0}}exp \{f({\textbf{y}};\hat{\varvec{\theta }})\}},\ \ for all {\textbf{y}}\in B_{0}, \end{aligned}$$

where the fraction on the right-hand side is an estimate of $P({\textbf{Y}}={\textbf{y}}\!\mid \!B_{0})$, for all ${\textbf{y}}\in B_{0}$. To measure the validity of $f({\textbf{y}};\hat{\varvec{\theta }})$ in the subpopulation defined by $B_{0}$, the mean-squared error given by $\sum _{{\textbf{y}}\in B_{0}}(n_{{\textbf{y}}0}-{\hat{n}}_{{\textbf{y}}})^{2}/|B_{0}|$, where $|B_{0}|$ is the cardinality of $B_{0}$, can be used. An alternative estimate of $\varvec{\theta }$, however, is given by the value of $\varvec{\theta }$ that maximizes the likelihood function $L_{B\cup B_{0}}$ in the training sample. Let $\bar{\varvec{\theta }}$ be the value of $\varvec{\theta }$ that maximizes $L_{B\cup B_{0}}$ in the training sample, then the predicted frequency of score pattern ${\textbf{y}}\in B_{0}$ in the validation sample, can alternatively be obtained by replacing $\hat{\varvec{\theta }}$ with $\bar{\varvec{\theta }}$ and $B_{0}$ with $B\cup B_{0}$ in the right-hand side of Eq. 36.

To obtain insights on how a special case of the general subpopulation model in Eq. 5 will generalize to an independent data set when no validation data set is available, *g*-fold cross-validation can be employed using the elements $B_{1},\ldots ,B_{g}$ of a partition of *B* as *g* folds. Since the result in Theorem 1 only requires that $B\subseteq S$, the result of Theorem 1 also applies to the training subset $D_{p}=B\backslash B_{p}$, that is,

37 $$\begin{aligned} P({\textbf{Y}}={\textbf{y}}\!\mid \!D_{p})=\frac{{\textbf{1}}_{D_{p}}({\textbf{y}})exp \{f({\textbf{y}};\varvec{\theta })\}}{\displaystyle \sum _{{\textbf{y}}\in D_{p}}exp \{f({\textbf{y}};\varvec{\theta })\}},\ \ for all {\textbf{y}}\in A . \end{aligned}$$

Let $\tilde{\varvec{\theta }}_{\!p}$ be the value of $\varvec{\theta }$ that maximizes the likelihood function $L_{D_{p}}=\prod \limits _{h\ne p}L_{h}$. The predicted frequency of the left-out score pattern ${\textbf{y}}\in B_{p}$, based on $D_{p}$, can then be given by

38 $$\begin{aligned} {\tilde{n}}_{{\textbf{y}}}=n\frac{exp \{f({\textbf{y}};\tilde{\varvec{\theta }}_{p})\}}{\displaystyle \sum _{{\textbf{y}}\in B}exp \{f({\textbf{y}};\tilde{\varvec{\theta }}_{p})\}},\ \ for all {\textbf{y}}\in B_{p}, \end{aligned}$$

where $n=\sum _{{\textbf{y}}\in O}n_{{\textbf{y}}}$. An alternative predicted frequency of the left-out score pattern ${\textbf{y}}\in B_{p}$ can be obtained by replacing *n* with $n_{p}$ and *B* with $B_{p}$ in the right-hand side of Eq. 38. In either case, the predicted frequency can be determined for all ${\textbf{y}}\in B$. Subsequently, the mean-squared error given by $\sum _{{\textbf{y}}\in B}(n_{{\textbf{y}}}-{\tilde{n}}_{{\textbf{y}}})^{2}/|B|$ can be calculated.

## Simulation Study

In this simulation study, the asymptotic bias and efficiency of the maximum likelihood estimators in $\hat{\varvec{\Theta }}_{B}$ and the usefulness of the goodness-of-fit tests are studied. Concerning the test statistics, special interest is in the sample sizes for which their distributions are satisfactorily close to the theoretical asymptotic chi-square sampling distributions. The goodness-of-fit tests are the Pearson chi-square test (CS), the likelihood ratio test of the hypothesized (sub)population model against the saturated (sub)population model (LR$_{\textrm{s}}$), the likelihood ratio test of the hypothesized (sub)population model against its first generalization (LR$_{1}$), the likelihood ratio test of the hypothesized (sub)population model against its second generalization (LR$_{2}$), and the likelihood ratio test of the first generalization against the second generalization (LR$_{12}$).

To study the bias and efficiency of the maximum likelihood estimators, the average absolute approximate bias, the average approximate mean square error, and the average approximate variance over estimates have been calculated. In addition, the average of squared estimated standard errors has been calculated. To study the usefulness of the tests, rejection rates have been calculated. The calculations have been carried out under six sample sizes, that is, $n\in \{250,500,750,1000,5000,10{,}000\}$ and two numbers of variables, that is, $k\in \{5,10\}$. The number of elements of the partition of the set of observed score patterns is fixed to two. For convenience, score patterns have been randomly assigned to the elements of the partition.

The R program (R Core Team, 2020) has been used to generate data under two models for binary variables. In the first data generation model, $f({\textbf{y}})=\sum _{i=1}^{k}\beta _{i}x_{i}+\sigma _{2}{\textbf{1}}'{\textbf{y}}({\textbf{1}}'{\textbf{y}}-1)/2$. This model is a simple special case of both the extended Rasch model and the Ising model and is called the conditional normal extended Rasch (cn-ER) model. The second data generation model is the more complex Ising model, where $f({\textbf{y}})=\sum _{i=1}^{k}\beta _{i}x_{i}+\sum _{i<j}x_{i}x_{j}\sigma _{ij}$. In both data generation models, the vector $(\beta _{1},\ldots ,\beta _{k})$ is fixed to $(-3.5,-2.5,-1.5,-0.5,0.5)$, for $k=5$, and to $(-5.0,-4.5,-4.0,-3.5,-3.0,-2.5,-2.0,-1.5,-1.0,-0.5)$, for $k=10$. In the cn-ER model, the parameter $\sigma _{2}$ is set to .63. In the Ising model, $\sigma _{ij}$, for all $i<j$, is randomly sampled from a normal distribution with mean 30 and standard deviation 5, for $k=5$, and from a normal distribution with mean 30 and standard deviation 1, for $k=10$.

Data have been generated under two support conditions: $S=A$ and $S\subset A$. In the case of $S=A$, the number of observable score patterns is $2^5=32$, for $k=5$, and $2^{10}=1024$, for $k=10$. In conditions in which $S\subset A$, an arbitrary substantial number of score patterns that showed low probabilities in the case of $S=A$, have been excluded from *S*. In the case of $S\subset A$, $k=5$, and the cn-ER model, the cardinality of *S* is 15. In the case of $S\subset A$, $k=5$, and the Ising model, the cardinality of *S* is 19. In the case of $S\subset A$, $k=10$, and the cn-ER model, the cardinality of *S* is 202. In the case of $S\subset A$, $k=10$, and the Ising model, the cardinality of *S* is 198.

In each of the 48 conditions (2 numbers of variables $\times$ 6 sample sizes $\times$ 2 models $\times$ 2 support conditions), 10,000 data sets have been randomly generated. For each data set, the saturated model, the data generation model, its first generalization, and its second generalization have been fitted to the data twice, once using $B=O$ and second using $B=A$. All models have been fitted to the data using the R function glm(). The five tests have also been carried out twice, once using $B=O$ and second using $B=A$. The nominal level of significance was set at .05, for all tests. In each condition, for each test a rejection rate has been calculated. The rejection rate of a test is the number of times the null hypothesis model has been rejected in favor of the alternative hypothesis model divided by 10,000. Most calculated rejection rates are approximate type I error rates and should be close to the nominal level of significance. In the case of $S\subset A$ and $B=A$, however, calculated rejection rates are approximate power values.

In nearly all conditions, the approximate variance of the estimator over estimates and the average of the squared estimated standard errors are the same up to three decimal places. Only now and then (for lower sample sizes) they differ in the third decimal. Therefore, only the approximate variance of the estimator over estimates is reported.

**Table 1:** Median and range of the cardinality of *O*, averages of absolute approximate bias, approximate mean square error, and approximate variance of the estimator, for $S=A$.

*k*	Model	*n*	Median |*O*|	Range |*O*|	$B=O$			$B=A$		
					Bias	MSE	$var({\hat{\Theta }})$	Bias	MSE	$var({\hat{\Theta }})$
5	cn-ERM	250	24	[18, 30]	0.115	0.064	0.043	0.009	0.046	0.046
		500	27	[22, 32]	0.047	0.026	0.022	0.004	0.023	0.023
		750	28	[23, 32]	0.027	0.016	0.015	0.003	0.015	0.015
		1000	29	[25, 32]	0.019	0.012	0.011	0.003	0.011	0.011
		5000	32	[29, 32]	0.001	0.002	0.002	0.000	0.002	0.002
		10,000	32	[30, 32]	0.000	0.001	0.001	0.000	0.001	0.001
	Ising	250	22	[15, 29]	0.246	0.446	0.245	0.050	0.264	0.261
		500	25	[18, 31]	0.122	0.183	0.124	0.040	0.147	0.144
		750	26	[21, 32]	0.077	0.106	0.081	0.026	0.100	0.098
		1000	27	[22, 32]	0.056	0.073	0.062	0.022	0.074	0.072
		5000	31	[27, 32]	0.005	0.013	0.013	0.004	0.013	0.013
		10,000	32	[29, 32]	0.001	0.006	0.006	0.002	0.006	0.006
10	cn-ERM	250	104	[84, 126]	1.162	2.046	0.029	0.015	0.043	0.043
		500	154	[129, 183]	0.804	0.992	0.017	0.007	0.021	0.021
		750	190	[157, 219]	0.627	0.606	0.012	0.005	0.014	0.014
		1000	218	[187, 249]	0.517	0.413	0.009	0.003	0.010	0.010
		5000	415	[379, 450]	0.149	0.035	0.002	0.001	0.002	0.002
		10,000	512	[474, 548]	0.081	0.011	0.001	0.000	0.001	0.001
	Ising	250	105	[87, 131]	0.687	1.326	0.311	0.083	0.353	0.341
		500	153	[127, 181]	0.516	0.747	0.130	0.044	0.168	0.164
		750	185	[156, 216]	0.427	0.522	0.085	0.031	0.110	0.108
		1000	211	[182, 239]	0.366	0.395	0.063	0.024	0.083	0.081
		5000	389	[354, 426]	0.125	0.060	0.013	0.005	0.015	0.015
		10,000	479	[444, 514]	0.070	0.023	0.007	0.002	0.008	0.008

The asymptotic bias and efficiency results for $S=A$ are given in Table 1. The results in Table 1 show that in the case of $S=A$, the averages of the absolute approximate bias, the approximate mean square error, and the approximate variance of the estimator, all tend to zero as the sample size increases, irrespective of the number of variables, model complexity, and the selection of *B*. As expected in the case of $S=A$, the averages of the absolute approximate bias and the approximate mean square error are in general higher for $B=O$ than for $B=A$. Note that in the case of $k=10$, the averages of the absolute approximate bias and the approximate mean square error in the case of $B=O$ are based on much smaller numbers of score patterns than in the case of $B=A$ because the maximum cardinality of *O* is 548. On the other hand, the averages of the approximate variance of the estimator do not seem to depend on the choice of *B*.

**Table 2:** Approximate type I error or rejection rates of the CS, LR$_{\textrm{s}}$, LR$_{1}$, LR$_{2}$, and LR$_{12}$ tests at the .05 nominal level of significance, for $S=A$.

*k*	Model	*n*	$B=O$					$B=A$				
			CS	LR$_{\textrm{s}}$	LR$_{1}$	LR$_{2}$	LR$_{12}$	CS	LR$_{\textrm{s}}$	LR$_{1}$	LR$_{2}$	LR$_{12}$
5	cn-ER	250	.048	.019	.036	.049	.036	.074	.035	.060	.052	.062
		500	.052	.023	.042	.049	.041	.068	.054	.053	.048	.052
		750	.051	.026	.044	.048	.045	.060	.058	.053	.054	.051
		1000	.050	.026	.043	.047	.044	.063	.068	.054	.049	.056
		5000	.050	.046	.050	.048	.052	.051	.056	.050	.046	.050
		10,000	.052	.052	.048	.049	.048	.052	.059	.053	.049	.055
	Ising	250	.014	.009	.009	.027	.010	.077	.015	.031	.058	.032
		500	.029	.015	.017	.043	.017	.080	.033	.048	.053	.048
		750	.040	.023	.025	.045	.023	.074	.040	.052	.051	.052
		1000	.048	.031	.033	.051	.033	.065	.042	.051	.054	.052
		5000	.038	.029	.032	.048	.032	.056	.066	.060	.051	.061
		10,000	.046	.042	.044	.053	.042	.051	.062	.060	.050	.061
10	cn-ER	250	.000	.000	.026	.050	.020	.244	.000	.053	.055	.052
		500	.032	.000	.086	.075	.074	.245	.000	.050	.052	.048
		750	.185	.000	.118	.080	.106	.249	.000	.056	.054	.054
		1000	.382	.000	.130	.082	.118	.249	.000	.051	.052	.051
		5000	.953	.010	.114	.072	.110	.245	.000	.047	.044	.048
		10,000	.966	.011	.088	.068	.084	.222	.000	.048	.047	.047
	Ising	250	.001	.000	.000	.036	.000	.130	.000	.186	.052	.187
		500	.010	.000	.000	.056	.000	.181	.000	.099	.054	.099
		750	.036	.000	.005	.066	.004	.190	.000	.082	.050	.083
		1000	.087	.000	.011	.068	.011	.211	.000	.074	.052	.075
		5000	.736	.001	.061	.062	.060	.217	.000	.055	.048	.056
		10,000	.862	.003	.068	.057	.066	.217	.000	.049	.052	.049

All calculated rejection rates for $S=A$ are approximate type I error rates. These approximate type I error rates are given in Table 2. The results in Table 2 show that in the case of $k=5$, none of the rejection rates is unacceptably higher than .05 and most of the rejection rates are close to .05. The following can be said about the rejection rates for $S=A$ and $k=10$. The rejection rate of the CS test is under most conditions too high. The rejection rate of the LR$_{\textrm{s}}$ test is too low under all conditions. In the case of $B=A$ and the cn-ER model, the rejection rates of the LR$_{1}$ and LR$_{12}$ tests are close to .05. In the case of the Ising model, the rejection rates of the LR$_{1}$ and LR$_{12}$ tests tend to .05 as the sample size increases. In the case of the cn-ER model, the rejection rates of the LR$_{1}$ and LR$_{12}$ tests are under most conditions too high if $B=O$. The rejection rate of the LR$_{2}$ test is close to .05 under all conditions.

**Table 3:** Median and range of the cardinality of *O*, averages of absolute approximate bias, approximate mean square error, and approximate variance of the estimator, for $S\subset A$.

*k*	Model	*n*	Median |*O*|	Range |*O*|	$B=O$			$B=A$		
					Bias	MSE	$var({\hat{\Theta }})$	Bias	MSE	$var({\hat{\Theta }})$
5	cn-ERM	250	15	[13, 15]	0.014	0.072	0.072	0.438	0.381	0.054
		500	15	[14, 15]	0.006	0.036	0.036	0.432	0.344	0.026
		750	15	[15, 15]	0.005	0.024	0.024	0.427	0.329	0.018
		1000	15	[15, 15]	0.004	0.017	0.017	0.426	0.322	0.013
		5000	15	[15, 15]	0.002	0.003	0.003	0.425	0.310	0.003
		10,000	15	[15, 15]	0.000	0.002	0.002	0.424	0.307	0.001
	Ising	250	18	[14, 19]	0.110	0.320	0.265	0.191	0.324	0.256
		500	19	[16, 19]	0.057	0.184	0.163	0.187	0.215	0.136
		750	19	[17, 19]	0.054	0.133	0.114	0.182	0.172	0.093
		1000	19	[17, 19]	0.052	0.104	0.085	0.178	0.144	0.069
		5000	19	[19, 19]	0.045	0.040	0.015	0.163	0.076	0.013
		10,000	19	[19, 19]	0.044	0.033	0.008	0.163	0.068	0.006
10	cn-ERM	250	90	[71, 112]	0.820	1.026	0.035	0.345	0.224	0.045
		500	124	[102, 146]	0.457	0.335	0.020	0.335	0.193	0.023
		750	145	[126, 166]	0.293	0.145	0.015	0.333	0.183	0.015
		1000	159	[140, 177]	0.205	0.076	0.012	0.332	0.179	0.011
		5000	201	[195, 202]	0.005	0.003	0.003	0.328	0.166	0.002
		10,000	202	[200, 202]	0.001	0.001	0.001	0.328	0.164	0.001
	Ising	250	93	[74, 110]	0.521	1.028	0.358	0.296	0.570	0.397
		500	126	[107, 144]	0.319	0.491	0.166	0.291	0.397	0.207
		750	145	[122, 166]	0.223	0.326	0.115	0.280	0.323	0.142
		1000	158	[137, 174]	0.169	0.251	0.089	0.273	0.283	0.107
		5000	190	[190, 198]	0.045	0.112	0.022	0.247	0.162	0.020
		10,000	198	[195, 198]	0.044	0.100	0.011	0.244	0.149	0.010

The asymptotic bias and efficiency results for $S\subset A$ are given in Table 3. The results in Table 3 show that in the case of $S\subset A$, the averages of the absolute approximate bias, the approximate mean square error, and the approximate variance of the estimator, all tend to zero as the sample size increases, irrespective of the number of variables and model complexity, if $B=O$. In the case of $B=A$, only the average of the approximate variance of the estimator seems to tend to zero as the sample size increases, irrespective of the number of variables and model complexity. In the case of $k=5$, the averages of the absolute approximate bias are all smaller for $B=O$ than for $B=A$. In the case of $k=10$, the averages of the absolute approximate bias are higher for $B=O$ than for $B=A$ if the sample size is less than 750 but smaller for $B=O$ than for $B=A$ if the sample size is greater than 500.

**Table 4:** Rejection rates of the CS, LR$_{\textrm{s}}$, LR$_{1}$, LR$_{2}$, and LR$_{12}$ tests at the .05 nominal level of significance, for $S\subset A$.

*k*	Model	*n*	$B=O$					$B=A$				
			CS	LR$_{\textrm{s}}$	LR$_{1}$	LR$_{2}$	LR$_{12}$	CS	LR$_{\textrm{s}}$	LR$_{1}$	LR$_{2}$	LR$_{12}$
5	cn-ER	250	.045	.051	.056	.049	.058	.063	.199	.505	.136	.487
		500	.046	.051	.049	.051	.049	.726	.992	.822	.205	.800
		750	.049	.053	.051	.054	.050	.997	1.000	.908	.273	.892
		1000	.048	.050	.048	.049	.043	1.000	1.000	.946	.321	.932
		5000	.052	.053	.050	.046	.054	1.000	1.000	.999	.609	.998
		10,000	.053	.053	.055	.050	.053	1.000	1.000	1.000	.705	.999
	Ising	250	.022	.019	.019	.052	.018	.001	.002	.007	.077	.007
		500	.036	.037	.037	.048	.038	.004	.024	.066	.109	.061
		750	.041	.048	.047	.054	.046	.018	.146	.279	.133	.261
		1000	.046	.052	.051	.056	.052	.077	.507	.643	.165	.599
		5000	.050	.052	.053	.054	.052	1.000	1.000	.991	.427	.991
		10,000	.048	.048	.048	.055	.049	1.000	1.000	.994	.566	.992
10	cn-ER	250	.000	.000	.007	.033	.005	.000	.000	.072	.058	.071
		500	.000	.000	.015	.045	.014	.000	.000	.094	.064	.093
		750	.000	.000	.024	.046	.024	.000	.000	.115	.075	.111
		1000	.000	.000	.025	.041	.028	.000	.000	.143	.079	.138
		5000	.032	.032	.048	.052	.049	.000	.000	.640	.194	.614
		10,000	.050	.062	.052	.050	.052	.000	1.000	.895	.294	.877
	Ising	250	.000	.000	.000	.026	.000	.000	.000	.189	.061	.186
		500	.000	.000	.000	.036	.000	.000	.000	.153	.062	.152
		750	.000	.000	.000	.038	.000	.000	.000	.180	.070	.177
		1000	.000	.000	.001	.038	.001	.000	.000	.225	.073	.222
		5000	.026	.030	.040	.050	.038	.000	.000	.968	.158	.966
		10,000	.048	.059	.051	.049	.051	.000	.000	1.000	.244	1.000

All calculated rejection rates for $S\subset A$ are given in Table 4. The results in Table 4 show that in the case of $B=O$, none of the rejection rates is unacceptably higher than .05. In the case of $B=O$ and $k=5$, most of the rejection rates are close to .05. In the case of $B=O$ and $k=10$, most rejection rates are less than .05 but are closer to .05 as the sample size increases. In the case of $B=A$, the rejection rates are approximate power values instead of approximate type I error rates because in these conditions the support of the probability distribution of the categorical variables is misspecified. As could be expected, the results in Table 4 show that for $B=A$ and $k=5$, all rejection rates are closer to one as the sample size increases. Under these conditions, the LR$_{2}$ test seems to be the least powerful. In the case of $B=A$ and $k=10$, the rejection rates of the new likelihood ratio tests are also closer to one if the sample size increases. Out of the new likelihood ratio test the LR$_{2}$ is again the least powerful under these conditions. In the case of $B=A$ and $k=10$, the rejection rates of the CS and LR$_{\textrm{s}}$ tests are all close to zero, except one.

It can be concluded that the LR$_{2}$ test performs better than the other tests if either $B=O$ or the support has been correctly specified. Under these conditions, unlike the rejection rates of the other tests, the rejection rate of the LR$_{2}$ test does not seem to be affected by the number of variables, model complexity, and sample size. Furthermore, it can be concluded that if the support of the probability distribution of the categorical variables is misspecified, then parameter estimates contain more bias and under many conditions, correctly specified $f({\textbf{y}};\varvec{\theta })$ is more often rejected than it should be using one of the tests.

In addition to the asymptotic performance of the tests under correctly specified conditions, it is also of interest to compare the power of the tests under misspecified $f({\textbf{y}};\varvec{\theta })$ in conditions in which all tests have type I error rates close to the nominal level of significance. According to the previous results, a fair comparison of the power of the tests is possible when data are generated under the Ising model, $k=5$, and $S=A$, and the cn-ER model is fitted to the data using both $B=O$ and $B=A$. For the same sample sizes as before 10,000 data sets have been generated with the same parameter values and all tests have been carried out for each data set under both $B=O$ and $B=A$. Rejection rates have been calculated and are given in Table 5.

**Table 5:** Approximate power values of the CS, LR$_{\textrm{s}}$, LR$_{1}$, LR$_{2}$, and LR$_{12}$ tests at the .05 nominal level of significance, for $S=A$.

*k*	Model		*n*	$B=O$					$B=A$				
	True	Fitted		CS	LR$_{\textrm{s}}$	LR$_{1}$	LR$_{2}$	LR$_{12}$	CS	LR$_{\textrm{s}}$	LR$_{1}$	LR$_{2}$	LR$_{12}$
5	Ising	cn-ER	250	.516	.393	.323	.159	.283	.456	.328	.337	.158	.298
			500	.876	.827	.638	.254	.568	.810	.786	.616	.248	.542
			750	.974	.963	.809	.333	.734	.956	.956	.788	.330	.707
			1000	.996	.995	.900	.396	.829	.994	.995	.884	.383	.810
			5000	1.000	1.000	.999	.680	.996	1.000	1.000	.999	.695	.996
			10,000	1.000	1.000	1.000	.770	1.000	1.000	1.000	1.000	.779	1.000

The results in Table 5 show that the CS test is the most powerful test and the LR$_{2}$ test is the least powerful test, irrespective of sample size and choice of *B*. In addition, the results show that all tests have higher approximate power values in using $B=O$ instead of $B=A$, for most sample sizes.

## An Empirical Data Example

The data in this example are the responses of 493 adolescents to seven items that are intended to measure nonaggressive antisocial behavior (Dekovic, 2003). The adolescents have been asked to indicate how often they have committed the antisocial act given by each item in the last twelve months. The responses are coded as 0 = *never*, 1 = *once*, 2 = *two or three times*, 3 = *four to ten times*, and 4 = *more than ten times*. The cardinality of *A* is $5^7=78125$. The number of observed score patterns is 267 (the cardinality of *O*). The extended partial credit model in Eq. 4 is fitted to the data twice, once using $B=A$ (assuming $S=A$) and second using $B=O$. The number of parameters in this model is $\sum \nolimits _{i=1}^{k}m_{i}+k-1=\sum \nolimits _{i=1}^{7}4+7-1=7\cdot 4+6=34$. The parameter estimation results are given in Table 6.

**Table 6:** Estimation results under the extended partial credit model in Eq. 4 for the Dekovic (2003) data.

Parameter	$B=A$		$B=O$	
	Estimate	SE	Estimate	SE
$\beta _{11}$	$-$ 0.995	0.173	$-$ 1.215	0.180
$\beta _{12}$	$-$ 1.415	0.305	$-$ 2.552	0.330
$\beta _{13}$	$-$ 3.162	0.469	$-$ 4.554	0.490
$\beta _{14}$	$-$ 4.657	0.624	$-$ 6.527	0.642
$\beta _{21}$	$-$ 1.903	0.189	$-$ 2.026	0.197
$\beta _{22}$	$-$ 2.914	0.334	$-$ 3.716	0.346
$\beta _{23}$	$-$ 5.430	0.515	$-$ 5.713	0.528
$\beta _{24}$	$-$ 7.597	0.689	$-$ 7.586	0.692
$\beta _{31}$	$-$ 3.271	0.225	$-$ 2.285	0.229
$\beta _{32}$	$-$ 5.076	0.384	$-$ 4.115	0.385
$\beta _{33}$	$-$ 7.904	0.614	$-$ 6.133	0.658
$\beta _{34}$	$-$ 10.384	0.825	$-$ 6.917	0.825
$\beta _{41}$	$-$ 2.299	0.205	$-$ 2.080	0.202
$\beta _{42}$	$-$ 2.985	0.338	$-$ 3.582	0.345
$\beta _{43}$	$-$ 4.930	0.505	$-$ 5.438	0.516
$\beta _{44}$	$-$ 6.511	0.662	$-$ 7.247	0.674
$\beta _{51}$	$-$ 2.850	0.214	$-$ 2.466	0.214
$\beta _{52}$	$-$ 4.111	0.356	$-$ 3.973	0.355
$\beta _{53}$	$-$ 6.418	0.538	$-$ 5.643	0.549
$\beta _{54}$	$-$ 8.300	0.702	$-$ 7.666	0.731
$\beta _{61}$	$-$ 2.633	0.202	$-$ 2.272	0.203
$\beta _{62}$	$-$ 4.500	0.365	$-$ 3.663	0.383
$\beta _{63}$	$-$ 7.219	0.573	$-$ 5.692	0.605
$\beta _{64}$	$-$ 10.032	0.813	$-$ 7.582	0.873
$\beta _{71}$	$-$ 3.081	0.213	$-$ 2.242	0.216
$\beta _{72}$	$-$ 5.969	0.440	$-$ 4.038	0.475
$\beta _{73}$	$-$ 9.571	0.876	$-$ 6.494	1.082
$\beta _{74}$	$-$ 12.366	1.229	$-$ 8.643	1.700
$\sigma _{2}$	0.345	0.158	0.749	0.165
$\sigma _{3}$	$-$ 0.124	0.126	$-$ 0.336	0.136
$\sigma _{4}$	0.069	0.074	0.152	0.084
$\sigma _{5}$	$-$ 0.031	0.031	$-$ 0.057	0.037
$\sigma _{6}$	0.009	0.008	0.015	0.010
$\sigma _{7}$	$-$ 0.001	0.001	$-$ 0.002	0.001

**Table 7:** Goodness-of-fit test results for the extended partial credit model in Eq. 4 for the Dekovic (2003) data.

Statistic	$B=A$			$B=O$		
	Value	df	*p* value	Value	df	*p* value
CS	62,415.090	78,090	1.000	105.467	232	1.000
LR$_{\textrm{s}}$	1569.898	78,090	1.000	97.212	232	1.000
LR$_{1}$	43.797	35	0.146	15.745	32	0.993
LR$_{2}$	0.601	1	0.348	0.565	1	0.452
LR$_{12}$	43.196	34	0.134	15.180	31	0.992

The goodness-of-fit test results are given in Table 7. Using $B=O$, some parameters of the first generalization of the subpopulation partial credit model could not be estimated due to too little observations for some categories. As a consequence, the degrees of freedom for the LR$_{1}$ and LR$_{12}$ tests are smaller than in case of $B=A$. According to all test results in Table 7, the extended partial credit model cannot be rejected at the 5 percent level of significance.

## Discussion

Using the conditional probability distribution of the categorical variables given the set *O* of observed score patterns, in principle, only requires a random sample from the subpopulation defined by *O*. In practice, however, a random sample from the total population is still required to guarantee that the sample is a random sample from the subpopulation defined by *O* because *O* is only known after the sample has been drawn. Not needing, in principle, a random sample from the total population is characteristic of fixed effects regression models. The model for the subpopulation defined by the set of all observed score patterns can also be seen as a fixed effects regression model, that is, a regression model where the conditional distribution of the random outcome frequency $N_{{\textbf{y}}}$ given fixed ${\textbf{y}}$ is a Poisson distribution, for all ${\textbf{y}}\in O$. Note that if the observed sample frequencies are the observations of independent Poisson random frequencies and the mean of random frequency $N_{{\textbf{y}}}$ is $\lambda _{{\textbf{y}}}=\alpha \,exp \{f({\textbf{y}})\}$, for all ${\textbf{y}}\in O$, then the likelihood function is given by

39 $$\begin{aligned} \prod _{{\textbf{y}}\in O}\frac{\lambda _{{\textbf{y}}}^{n_{{\textbf{y}}}}exp (-\lambda _{{\textbf{y}}})}{n_{{\textbf{y}}}!}=\alpha ^{n}exp \!\left[ \sum \limits _{{\textbf{y}}\in O}n_{{\textbf{y}}}f({\textbf{y}})-\alpha \sum \limits _{{\textbf{y}}\in O}exp \{f({\textbf{y}})\}\right] \!/\!\left( \prod \limits _{{\textbf{y}}\in O}n_{{\textbf{y}}}!\right) \end{aligned}$$

and the maximum likelihood estimate of the normalizing constant $\alpha$ equals $n/\sum _{{\textbf{y}}\in O}exp \{{\hat{f}}({\textbf{y}})\}$, where ${\hat{f}}({\textbf{y}})$ is the maximum likelihood estimate of $f({\textbf{y}})$ and the sum in the denominator is again only over all ${\textbf{y}}\in O$. Also note that the values that maximize the likelihood function in Eq. 39 are exactly the same as the values that maximize the likelihood function in Eq. 11, for $B=O$, because both likelihood functions yield the same estimating equations. Furthermore, it is a well-known fact that if the observed sample frequencies are the observations of independent Poisson random frequencies and the mean of random frequency $N_{{\textbf{y}}}$ is $\lambda _{{\textbf{y}}}=\alpha \,exp \{f({\textbf{y}})\}$, for all ${\textbf{y}}\in O$, then the conditional distribution of the random frequencies given $\sum _{{\textbf{y}}\in O}N_{{\textbf{y}}}=N=n$ is a multinomial distribution with parameters *n* and

40 $$\begin{aligned} \frac{\lambda _{{\textbf{y}}}}{\sum _{{\textbf{y}}\in O}\lambda _{{\textbf{y}}}}=\frac{exp \{f({\textbf{y}})\}}{\sum _{{\textbf{y}}\in O}exp \{f({\textbf{y}})\}},\ \ for all {\textbf{y}}\in O , \end{aligned}$$

which is exactly equal to the conditional probability in Eq. 5, for $B=O$. The multinomial likelihood function also yields the same estimating equations as the likelihood function in Eq. 11, for $B=O$, and therefore, also the same maximum likelihood estimates.

The null hypothesis tested by the goodness-of-fit tests for which the observed sample values of the test statistics are given by Formulas 29 and 34 is the hypothesis that the particular model used holds in the subpopulation defined by *B*. The null hypothesis tested by the goodness-of-fit test for which the observed sample value of the test statistic is given by Formula 35 is the hypothesis that the particular model used separately holds in each of the subpopulations defined by $B_{1},\ldots ,B_{g}$. The hypothesis that the model separately holds in each of the subpopulations defined by $B_{1},\ldots ,B_{g}$ is implied by the hypothesis that the model holds in the subpopulation defined by *B*. Since the null hypothesis that the model holds in the subpopulation defined by *B* is implied by the hypothesis that the model holds in the total population, rejection of any of the null hypotheses implies rejection of the total population model. If, on the other hand, the null hypotheses cannot be rejected, then the sample data do not provide evidence against the hypothesis that the model holds in total population. The hypothesis that the model holds in the total population, however, is not implied by any of the null hypotheses of the goodness-of-fit tests. This means that it might be that the specified model holds in the subpopulation defined by *B*, but not in the total population, or that the model separately holds in each of the subpopulations defined by $B_{1},\ldots ,B_{g}$ but not in the subpopulation defined by *B* nor in the total population. In such situations, the goodness-of-fit tests do not have any statistical power to reject the specified model in the total population.

The goodness-of-fit tests, but also the maximum likelihood procedure proposed, are not just applicable to the model for the subpopulation defined by the set *O* of all observed score patterns. The procedures can also be applied to the model for any subpopulation defined by either a superset of *O*, including the set of all theoretically possible score patterns, or a subset of *O*. Any superset of *O* can be assumed to be a subset of the true support of the probability distribution of the categorical variables. Assuming the set *A* to be the support, is one such an option. Assuming the support to include a specific proper superset of *O* not equal to *A*, is another option that already increases the computational efficiency relative to the all-inclusive support assumption. The problem then, however, is the selection of that specific proper superset of *O* and the possibility of wrongly including unobservable score patterns, that is, score patterns that are not in *S*. An example of a practical situation in which it is reasonable to exclude particular unobserved score patterns from the proper superset of *O* that is assumed to be a subset of the true support, is the situation in which the model of interest is the extended Rasch model for dichotomously scored items. In that situation, it is reasonable to exclude unobserved score patterns that are expected to be unobservable according to the deterministic Guttman model.

Any subset of the set of all observed score patterns defines a subpopulation with known support. The set of all observed score patterns defines the largest subpopulation with known support. An advantage of using the set *O* of all observed score patterns is that all data are used. Another possible subpopulation model that might be useful in practice is the model for the subpopulation defined by the set $\{{\textbf{y}}\in A\!\mid \!n_{{\textbf{y}}}\ge n_{0}\}$, where $n_{0}$ is a prespecified minimum sample frequency. In this case, observed score patterns with sample frequencies less than $n_{0}$ are not used. Possible negative consequences of choosing such a subpopulation are that the model parameters are estimated less precisely and that the hypothesized subpopulation model becomes underidentified.
